# Biochar from Co-Pyrolyzed Municipal Sewage Sludge (MSS): Part 2: Biochar Characterization and Application in the Remediation of Heavy Metal-Contaminated Soils

**DOI:** 10.3390/ma17153850

**Published:** 2024-08-03

**Authors:** Michael Biney, Mariusz Z. Gusiatin

**Affiliations:** Department of Environmental Biotechnology, Faculty of Geoengineering, University of Warmia and Mazury in Olsztyn, Sloneczna Str. 45G, 10-709 Olsztyn, Poland; michael.biney@uwm.edu.pl

**Keywords:** co-pyrolysis, municipal sewage sludge, biochar properties, pollutants, soil remediation

## Abstract

The disposal of municipal sewage sludge (MSS) from wastewater treatment plants poses a major environmental challenge due to the presence of inorganic and organic pollutants. Co-pyrolysis, in which MSS is thermally decomposed in combination with biomass feedstocks, has proven to be a promising method to immobilize inorganic pollutants, reduce the content of organic pollutants, reduce the toxicity of biochar and improve biochar’s physical and chemical properties. This part of the review systematically examines the effects of various co-substrates on the physical and chemical properties of MSS biochar. This review also addresses the effects of the pyrolysis conditions (temperature and mixing ratio) on the content and stability of the emerging pollutants in biochar. Finally, this review summarizes the results of recent studies to provide an overview of the current status of the application of MSS biochar from pyrolysis and co-pyrolysis for the remediation of HM-contaminated soils. This includes consideration of the soil and heavy metal types, experimental conditions, and the efficiency of HM immobilization. This review provides a comprehensive analysis of the potential of MSS biochar for environmental sustainability and offers insights into future research directions for optimizing biochar applications in soil remediation.

## 1. Introduction

Biochar, a carbon-rich by-product derived from the thermal decomposition of organic materials in an oxygen-limited environment, has emerged as a promising amendment for environmental management and agricultural improvement. In recent years, the focus has shifted toward innovative and sustainable sources for biochar production, such as municipal sewage sludge (MSS). Despite the numerous advantages of pyrolyzing MSS, the resulting biochar may exhibit high concentrations of certain pollutants, such as heavy metals (HMs), which can surpass those in the raw MSS. This increase is attributed to the reduction in mass during pyrolysis [1,2,3,4,5]. Although MSS contains varying concentrations of HMs, these levels can still be significant in environmental applications, increasing the environmental risk of MSS biochar and limiting its use, such as in soil amendments. It is important to note that lower concentrations of HMs in MSS are not always guaranteed, as the characteristics of MSS mainly depend on the chemical composition of the raw wastewater and can change during various stages of MSS processing in wastewater treatment plants.

Recent studies indicated that compared with biochar derived from MSS alone, co-pyrolysis of MSS with other feedstocks is an effective approach that enhances the properties of the biochar and improves the stability of the HMs [6]. As indicated in Part 1 [7] of this review, the reported co-substrates belong to both biomass (including forestry, agriculture, recycling waste, food residues, digestate, and microalgae) and non-biomass materials. Because these co-substrates vary in composition, they can have different effects on the physical and chemical properties of biochar under specific co-pyrolysis conditions. Therefore, co-pyrolysis can tailor the properties of biochar to specific needs by adjusting the feedstock composition. Co-pyrolysis of MSS can significantly influence the total HM concentration and their mobility in the resulting biochar. When MSS is mixed with other types of co-substrates, the overall HM concentrations can be diluted. Moreover, the interaction between MSS and different co-substrates can lead to the formation of more stable, less bioavailable metal forms. High heat and pressure during co-pyrolysis can also change the chemical species of HMs, binding them to the carbonaceous matrix or other elements present in the biochar, thus reducing their mobility and bioavailability. This is crucial for biochar intended as a soil amendment, where the lower mobility and bioavailability of HMs prevent environmental contamination. Co-pyrolysis can help mitigate these risks by enhancing the immobilization of HMs, making the resulting biochar more suitable for environmental applications.

Over the years, studies have demonstrated that the co-pyrolysis of MSS with forest and agricultural biomass such as bamboo sawdust, rice straw, and cotton stalk [8,9] can significantly reduce the bioavailability and leachability of HMs present in the resulting biochar [2] and enhance biochar properties such as the specific surface area (SSA), porosity, carbon (C) content, pH, cation exchange capacity (CEC) and nutrient levels [2,10]. For instance, Jin et al. observed that the co-pyrolysis of MSS and bamboo sawdust resulted in the transformation of HMs from the exchangeable fraction and reducible fraction to a potentially stable oxidizable fraction and residual fraction, thus reducing the toxicity of the HMs in the derived biochars [11]. Similarly, Sun et al. demonstrated the proficient performance of MSS and rice straw co-pyrolysis in increasing the yield of the resultant biochar, its alkalinity, its porosity and the stability of Cd [6]. Due to the wide variety of different co-substrates and the fact that co-pyrolysis can alter the properties of biochar, it is imperative to systematically analyze their impact on the physical and chemical properties of MSS biochar. This also includes investigating the effects on the content and stability of emerging pollutants. However, a systematic comparative review on the effects of feedstocks of different origin on the biochar properties and environmental risks associated with the presence of inorganic and organic pollutants has not yet been carried out.

In recent years, the utilization of MSS biochar has emerged as a promising and sustainable method for soil amendment and remediating HM-contaminated soils [12,13,14] despite the challenges posed by biochar containing HMs and other pollutants. However, with careful consideration of the pyrolysis conditions, such as the temperature, residence time, heating rate, carrier gas and gas flow rate, it is feasible to mitigate these challenges. The MSS biochar improves the nutrient supply of the soil with an effect that lasts for up to several years, especially in combination with mineral fertilizers, making it a viable option for improving soil fertility [15]. Besides enriching the soil chemistry by increasing the pH and levels of exchangeable calcium (Ca) and magnesium (Mg), the MSS biochar also improves physical properties like the soil stability [16,17]. Additionally, MSS biochar can promote plant growth and improve certain soil health parameters [18]. The introduction of MSS into soil has been known to positively change the activities and diversity of the soil microbial community, which plays a significant role in biochemical processes in soil. This in turn increases the soil respiratory activity that may result from biotic consumption of nutrients introduced by the organic material, abiotic sorption of CO_2_, or interaction between the MSS biochar and soil organic matter [19,20].

Biochar has good environmental sustainability for soil remediation, as it ensures the continued use of the soil and the protection of biodiversity. Another advantage is the low cost of biochar, its ease of preparation and its relatively high effectiveness [6]. Biochar from MSS could have an even higher efficiency in reducing the bioavailability of Cd than biochar produced from forest and agricultural biomass (manure, peanut shells, rice straw, wheat straw, wood, corn stalks and silver grass) [10]. Some authors indicate a higher immobilization efficiency of HMs in soil for MSS biochar from co-pyrolysis than for biochar from pyrolysis of MSS alone [21]. In addition, the remediation efficiency with MSS biochar from co-pyrolysis can be strongly influenced by different ratios between MSS and co-substates due to changes in the biochar properties [9].

Despite the intensive development of research on the pyrolysis and co-pyrolysis of MSS, reviews summarizing the effects of the different types of feedstocks on the physical and chemical properties of the resulting products, as well as analyses of the effects of such biochar on the remediation of HM-polluted soil, are lacking.

In this review, we aim to systematically investigate the effects of co-pyrolysis on the properties of MSS biochar and its application in the remediation of HM-contaminated soils. This review investigates how different co-substrates affect the physical and chemical properties of MSS biochar, and it explores the underlying processes and mechanisms that facilitate the immobilization of HMs. By summarizing recent studies on the use of MSS biochar for soil remediation, this review provides an overview of current applications, including conditions for effective HM immobilization. This review can serve as a valuable resource for researchers and practitioners by providing a holistic view of the benefits, mechanisms, and applications of co-pyrolyzed MSS biochar. Finally, this review suggests future research directions to optimize the use of MSS biochar in environmental management and soil remediation, demonstrating its potential for soil pollution control.

## 2. Properties of MSS Biochar from Co-Pyrolysis

Co-pyrolysis, similarly to pyrolysis alone, is a multistage, thermochemical process [7]. Co-pyrolysis mostly has a synergetic effect on MSS biochar, affecting its physical and chemical properties. The co-pyrolysis process helps reduce the ash content and increase the C content of the resultant biochar and also impacts the elemental composition of the resultant biochar. Biochars from MSS co-pyrolysis typically have a lower yield but higher pH and C content compared to biochar produced solely from MSS. Additionally, they often exhibit more aromatic structures, contributing to their enhanced properties [2,10,21]. Generally, the sulfur (S) and nitrogen (N) contents are lower in biochars produced via co-pyrolysis compared to those from MSS alone. This reduction can be attributed to the dilution effect caused by the addition of biomass feedstock, resulting in a decreased concentration of S and N in the final biochar product [2,10]. Furthermore, co-pyrolysis can facilitate the transformation of phosphorus (P) within the biochar, leading to a reduction in the total P content and an increase in the proportion of more readily plant-available forms, such as apatite P. This enhancement in plant-available P forms makes co-pyrolysis biochar a potentially valuable soil amendment [10]. Importantly, the co-pyrolysis process can significantly reduce the content and toxicity of HMs and organic pollutant in the resultant biochar. This reduction is achieved through the transformation of HMs into more stable forms, with a higher proportion of the HMs being bound in the residual fraction, which is a non-leachable and less bioavailable form [2,10].

**Figure 1:**
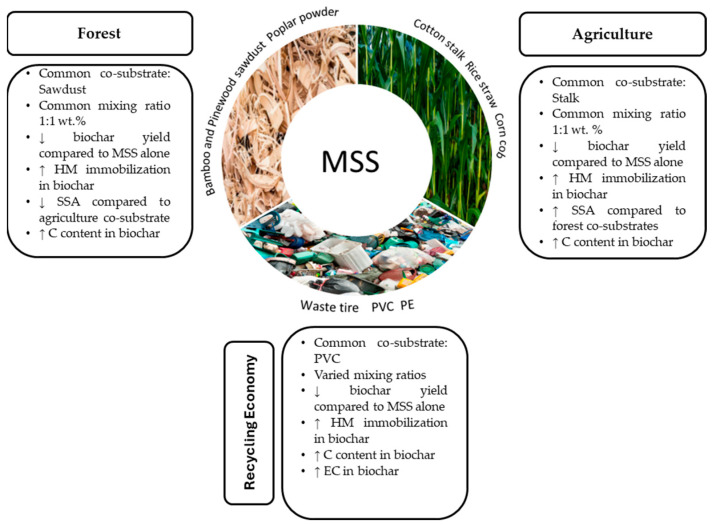
The main effects of the most common co-substrates on the properties of MSS biochar (↑ increase; ↓ decrease).

The main effects of most common co-substrates on the properties of MSS biochar are summarized in Figure 1. Table 1 provides a detailed assessment of the physical and chemical properties of MSS biochar compared to biochar produced by co-pyrolysis with different feedstocks. This comparison is important for a number of reasons. Firstly, it provides a clear comparative analysis highlighting the improvements in the physical and chemical properties of biochar through co-pyrolysis, which is crucial for evaluating the effectiveness of co-pyrolysis. Secondly, the data help to understand how different feedstocks and co-pyrolysis conditions affect the quality of the biochar. This knowledge is invaluable for future optimizations of the pyrolysis process aimed at improving the overall properties of the resulting biochar and enabling better production processes and technological advances in this area.

### 2.1. Physical Properties of Biochar from MSS Co-Pyrolysis

The co-pyrolysis of MSS with other feedstocks enhances the physical properties of the resultant biochar compared to MSS biochar alone. One significant improvement is the increase in the SSA of the co-pyrolysis biochar. The addition of the co-substrate helps reduce the ash content and increase the C content, contributing to a higher SSA value [3,32,33].

As indicated in Table 1, the physical properties of the MSS biochar from pyrolysis and co-pyrolysis are not always analyzed. Based on the available data, the SSA of biochar from MSS co-pyrolysis with forest and agriculture products ranges from 2.78 to 30.70 m^2^/g [11,26] and 4.39 to 122.90 m^2^/g [22,27], respectively, but it strongly depends on the type of co-substrate, the mixing ratios and the conditions of pyrolysis. For instance, the SSA of MSS and bamboo sawdust (1:1 *w*/*w*) at 400 °C was 2.8 m^2^/g [22] as compared to 15.8 m^2^/g [28] for MSS and cotton stalk (1:1 *w*/*w*) at the same pyrolysis temperature.

The pore structure of biochar resulting from the co-pyrolysis of MSS with other feedstocks is predominantly microporous, with average pore sizes typically <10 nm [3,33]. Similar to the SSA, studies have shown that the variation in the pore sizes also depends on the type of co-substrate, the mixing ratios and the conditions of pyrolysis. For instance, co-pyrolysis of MSS with cotton stalks at different temperatures resulted in an average pore volume (APV) ranging from 12.80 nm to 14.30 nm, deviating from the typical microporous ranges [28].

Moreover, co-pyrolysis influences the density of biochar by reducing it and increasing the porosity, making it beneficial for application as a soil amendment. Co-pyrolysis also influences the overall yield of the biochar, with the biochar from MSS co-pyrolysis typically exhibiting lower yields compared to MSS biochar alone. This is attributed to the co-pyrolysis process, which helps concentrate the C content of the biochar [32,33].

From this it can be concluded that the physical properties of MSS co-pyrolysis biochar depend primarily on the type of co-substrate, the mixing ratio and the pyrolysis temperature. However, the dependence on temperature is not linear, as an increase in the temperature of the co-pyrolysis biochar does not necessarily correlate with an increase in the SSA, APV and total pore volume (TPV), as shown in Table 1.

### 2.2. Chemical Properties of Biochar from MSS Co-Pyrolysis

The co-pyrolysis of MSS with other co-substrates results in biochar with enhanced chemical properties. These properties include a higher mineral content, lower S and N content, improved aromatic structure as well as reduced HM concentrations and ecotoxicity of the resultant biochar [32,34,35].

#### 2.2.1. Proximate Analysis

The proximate analysis of MSS co-pyrolysis biochar reveals a spectrum of compositional attributes, emphasizing its multifaceted utility in soil management and remediation. The increased fixed carbon (FC) content, low moisture and volatile matter (VM) content signify the biochar’s potential as a stable carbon sink and soil conditioner.

The VM content of MSS co-pyrolysis biochar is generally lower than that of MSS alone. This is attributed to the co-pyrolysis process, which promotes a higher degree of carbonization in the resultant biochar compared to biochar from MSS alone [34,36]. On average, the quantity of the ash content in MSS co-pyrolysis biochar is found to be reduced as compared to the MSS pyrolysis alone [10,21], and this depends on the specific co-substrate. For instance, it was observed that the ash content decreased from 61.9% to 41.5% when MSS was pyrolyzed alone as compared to when it was co-pyrolyzed with Salix (SX) at a mixing ratio of 1:1 ratio (MSS/SX) at 500 °C, respectively [25]. At the same temperature, a further decrease in the ash content was observed to 30.1% when the mixing ratio was decreased to 1:3 (MSS/SX). It is evident that mixing MSS with other biomass during co-pyrolysis reduces the high ash content of MSS. The FC content is increased in MSS co-pyrolysis biochar as compared to MSS alone [25,26,29,31]. This is evident because the lower VM content of MSS co-pyrolysis biochar indicates that volatile components are released from the pores during the pyrolysis process, making it more porous and increasing the FC content.

#### 2.2.2. Ultimate Analysis

Biochar produced from MSS co-pyrolysis typically has a higher C content compared to biochar from MSS alone. On average, the C content of MSS co-pyrolysis biochar ranges from 19.2 to 60.3%, whereas in MSS alone, it ranges from 6.9 to 32.4%, as shown in Table 1. This can be influenced by the mixing ratio and the H/C ratio (aromaticity) of the feedstock, as a lower aromaticity generally leads to a higher C content in the biochar [2].

In comparison to MSS alone, the introduction of bamboo sawdust as a co-substrate at a mixing ratio of 1:1 increased the C content from 21.9% to 31.2% at 400 °C. Furthermore, comparable outcomes were noted at 500 °C and 600 °C in relation to co-pyrolyzed biochar in the same proportion [11]. Comparing MSS biochar and co-pyrolyzed biochar with various co-substrates, such as pinewood sawdust, walnut shell, cotton stalks, rice straw and husk, and digested manure, the pattern of a higher C content remains constant. Hence, it can be inferred that co-pyrolysis increases the C content of the resultant biochar as compared to pyrolysis of MSS alone.

Similar to the C content, the H content tends to increase in MSS co-pyrolysis biochar compared to MSS biochar alone. Generally, the H and O content decreases relative to the C content in MSS co-pyrolysis biochar as the temperature rises. The elemental composition of MSS co-pyrolysis biochar shows a relatively low S content, ranging from 0.1 to 2.1%, compared to MSS alone. The general trend shows that the aromaticity and O/C ratio (polarity) decrease at higher temperatures, regardless of whether MSS co-pyrolysis or MSS alone is involved.

In the case of bamboo sawdust, the aromaticity and polarity showed different trends when comparing the MSS biochar with the co-pyrolyzed biochar. At temperatures of 400 °C and 500 °C, the aromaticity and polarity showed high values compared to the co-pyrolyzed biochar. However, at 600 °C, the co-pyrolyzed biochar showed an increased aromaticity value (as H/C) of 0.5 compared to the MSS biochar at 0.4. Nevertheless, at the same temperature of 600 °C, the polarity followed the previous trend of the MSS biochar, having a higher value than the co-pyrolyzed biochar, from a 2.9 to 1.6 molar ratio (as O/C). This trend was also observed for other co-substrates. For example, in the case of rice husk, the aromaticity of the MSS biochar at 400 °C was reported to be 1.0, while it was 0.8 for the co-pyrolyzed biochar at 400 °C, indicating a decrease in the aromatic content. In addition, the polarity of both the MSS biochar and the co-pyrolyzed biochar remained at a value of 0.3 at 400 °C. However, a slight change was observed at 700 °C, where the MSS biochar had a value of 0.1 and the co-pyrolyzed biochar had a value of 0.3. Conversely, there was a slight increase in aromaticity and polarity at 700 °C from an initial value of 0.2 for the MSS biochar to a final value of 0.3 for the co-pyrolyzed biochar and from 0.1 for the MSS biochar to 0.3 for the co-pyrolyzed biochar.

Upon comparison of the MSS co-pyrolysis biochar to MSS alone, a decrease in both aromaticity and polarity was observed. The significant influence of the mixing ratio of both co-substrates was clearly demonstrated. For instance, at a 1:1 ratio of MSS and MSS with walnut shell (WS) at 600 °C, the recorded values for aromaticity and polarity were 0.4 and 0.7, respectively. In contrast, the values at the same temperature were 0.7 and 1.4, and 0.3 and 0.4 for 3:1 (MSS/WS wt.%) and 1:3 (MSS/WS wt.%), respectively.

Co-pyrolysis is shown to enhance the physical and chemical properties of the resultant biochar. This includes an increase in the SSA and C content as well as reducing the ash content of the resultant biochar. The properties of co-pyrolysis biochar are mostly influenced by the type of co-substrates, the mixing ratio, and the pyrolysis temperature. With the consideration of these factors, enhanced biochars could be produced from MSS co-pyrolysis for soil amendment.

## 3. Effect of MSS Pyrolysis and Co-Pyrolysis on Micropollutants in Biochar

The use of MSS biochar as a soil amendment faces challenges due to the presence of micropollutants such as HMs, PAHs, PCDD/Fs, PFASs, and other contaminants in the biochar. It is worth noting that the toxicity of biochar is not influenced by the total content of pollutants but by their bioavailable or mobile fraction.

The type and concentration of micropollutants in MSS biochar can be adjusted significantly by selecting the appropriate process parameters for pyrolysis, including the pyrolysis temperature, type and flow rate of carrier gas, heating rate, and residence time, as well as a suitable co-substrate for co-pyrolysis. By optimizing these parameters, it is possible to reduce the levels of micropollutants in MSS biochar to a safe and acceptable level, making it a viable option for sustainable soil management and remediation.

### 3.1. HM in MSS Biochar

MSS typically contains HMs in the form of mineral salts (chlorates, carbonates, sulfates, phosphates), sulfides, hydroxides, and oxides. Upon exposure to pyrolysis, these HMs undergo a transformation process that leads to their conversion into more thermostable compounds compared to the original HMs. The reduced levels of certain HMs, particularly at high temperatures, indicate the possibility of partial defractionation and/or partial volatility of these elements at elevated temperatures, as observed by Zielinska et al. [37]. Similarly, Hossain et al. reported a similar phenomenon of a decreasing total content of Cr, Ni, and Pb during MSS pyrolysis at 700 °C [14].

The total HM concentrations in MSS biochar are often observed to be declining in the following order: Zn > Cu > Cr > Pb > Ni > Cd. A study conducted by Wang et al. on MSS biochar pyrolyzed within the range of 350–750 °C showed that the total concentration of Cd, Cr, Cu, Ni, Pb and Zn within MSS biochar increases proportionally with increasing temperatures [38]. Other studies have also indicated that when MSS is subjected to pyrolysis at moderate temperatures (400–600 °C), more than 90% of HMs are retained within the resulting biochar [11,39,40]. Li et al. observed that the biochar’s aromatization and SSA increased when the pyrolysis temperature was raised from 300 to 700 °C [4]. This resulted in HMs being trapped in the MSS biochar, but when the temperature was increased from 600 to 700 °C, the biochar’s residual Pb and Cd contents dramatically decreased from 90.72% to 79.27% and 91.26% to 86.48%, respectively. It is therefore reasonable to anticipate a large decrease in the bioavailability of HMs in MSS biochar as the temperature increases. Nonetheless, co-pyrolysis significantly decreases the overall HM concentration within the biochar, thereby facilitating the conversion from the mobile fraction to the stable fraction.

**Figure 2:**
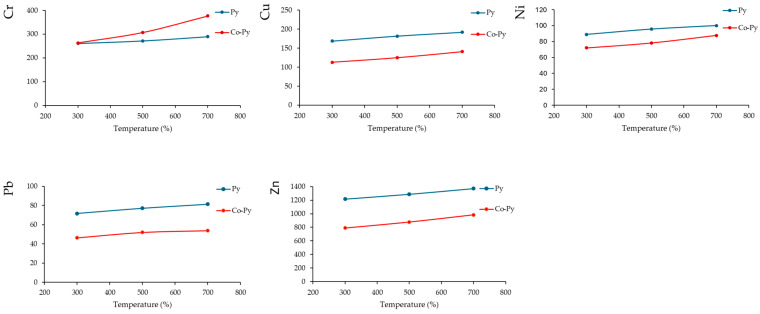
Total HM contents (in mg/kg) in MSS and MSS/reed (*Phragmites australis*) biochar (50:50 wt.%) [10]; Py means MSS pyrolysis, Co-Py means MSS co-pyrolysis.

The findings presented in Figure 2 confirm the significance of co-pyrolysis in decreasing the total HM content within biochar. The results show that the total HM content, with the exception of chromium (Cr), decreased when MSS was co-pyrolyzed with reed. The increase in Cr in the co-pyrolyzed biochar can be attributed to the relatively high concentration of Cr in the reed biomass (93.39 mg/kg) compared to MSS alone (182 mg/kg). The reduction in the HM content in co-pyrolyzed biochar indicates that co-pyrolysis of MSS offers a significant advantage in improving the quality and safety of the resulting biochar compared to biochar produced solely from MSS pyrolysis.

The HM bioavailability in biochar is usually determined based on single extraction protocols (e.g., test with DTPA) or with sequential extraction (e.g., BCR extraction procedure). Despite its complexity, sequential extraction offers a comprehensive assessment of the risks associated with HMs. This methodology facilitates the determination of both the bioavailable fraction and the fraction bound with varying bond strengths to different matrix components. A recent study by Bai et al. showed that MSS pyrolysis significantly immobilizes HMs by converting unstable, acid-soluble, reducible, and oxidizable fractions to stable residual fractions [41]. The co-pyrolysis of MSS with co-substrates such as cotton stalk, willow, or hazelnut shell has also been found to synergize HM immobilization more efficiently as compared to MSS biochar alone [2]. Kończak and Oleszczuk explored the effects of mixing MSS and willow on the available fraction of Cd, Co, Cr, Cu, Fe, Ni and Pb [42].

The study found that, at a ratio of 6:4 (MSS/willow), regardless of the temperature (500–700 °C), there was a decrease in the HM content in the co-pyrolyzed biochar as compared to the MSS biochar alone. When comparing the biochar produced with CO_2_ and N_2_ as the carrier gas, it was also found that the presence of CO_2_ led to a stronger immobilization of HMs in the biochar. However, the use of CO_2_ during co-pyrolysis decreased the available HM content from 9.0 to 100%, although the total HM content increased. In addition, Co, Pb and Cd were not identified as bioavailable in the co-pyrolyzed MSS biochar, suggesting that these HMs were fully bound in less available and unavailable forms. Moreover, a study conducted by Wang et al. revealed that the co-pyrolysis of MSS with food waste digestate (FWD) can significantly reduce the ecological risk assessment of HMs in biochars as compared to biochar obtained from single pyrolysis [40]. Co-pyrolysis significantly reduced the HM-associated ecological risk (potential ecological risk index lower than 15.51, indicating a low ecological risk from HM pollution) and phytotoxicity (germination index higher than 139.41%, indicating a relatively fast and high germination rate) of the mixed ratio ≥ 3:1 (FWD/MSS). The reduction in the ecological risk was attributed to the formation of more stable and less soluble chemical fractions, such as the oxidizable fraction and residual fraction. Interestingly, the formation of these fractions increases with the increase in the mixing ratio of FWD.

**Figure 3:**
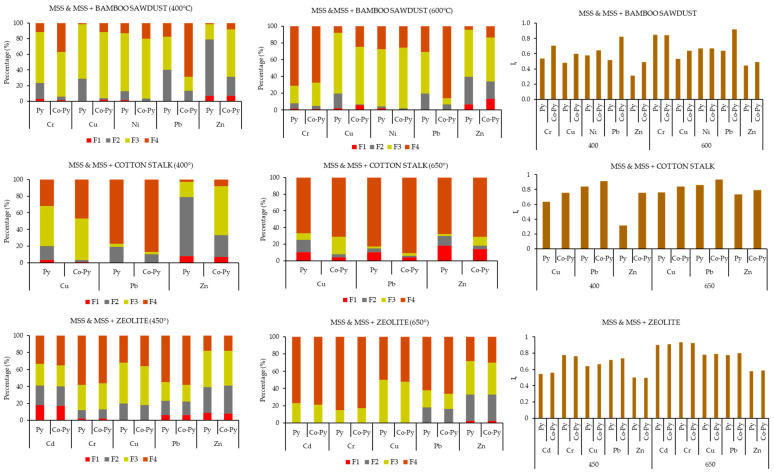
HM distribution and stability based on the reduced partition index (I_r_) in MSS biochar from pyrolysis and co-pyrolysis with selected co-substrates [23,28,43].

With HM distribution patterns, it is possible to access their stability using the reduced partition index (I_r_). In Figure 3, exemplary changes in the HM distribution for biochars obtained from pyrolysis and co-pyrolysis, and the resultant stability (as I_r_), for two opposite pyrolysis temperatures are compared. For this comparison, only selected co-substrates, such as bamboo sawdust, cotton stalks and zeolite, were chosen for evaluation, as the HM distribution is not always analyzed in biochars, making it difficult to consider other co-substrates. When comparing the fractionation and stability of HMs at moderate (400–450 °C) and higher (600–650 °C) temperatures for pyrolysis and co-pyrolysis biochar derived from MSS and the above co-substrates, a notable observation was made.

In the examination of MSS biochar and co-pyrolyzed biochar with bamboo sawdust, it is evident that the co-pyrolyzed biochar exhibited a significant increase in the F4 fraction for HMs compared to the MSS biochar alone at both 400 and 600 °C. This elevation was particularly pronounced for Pb, as the F4 fraction displayed a sharp increase at 400 °C and the most substantial increase at 600 °C. Additionally, Cr, Cu, Ni, and Zn also demonstrated heightened residual fractions, particularly at higher temperatures (600 °C). When assessing stability, it is noteworthy that Pb from co-pyrolysis biochar at 600 °C attained the highest stability, with an I_r_ value of 0.92, while Zn from MSS biochar at 400 °C registered the least stability, with an I_r_ value of 0.31 [11].

In addition, both MSS biochar and co-pyrolyzed biochar with cotton stalk displayed a similar pattern of an increased residual fraction (F4), particularly at higher temperatures (650 °C). Pb exhibited a notable increase in the F4 fraction, followed by Cu at 400 °C, and Zn demonstrated a marginal rise in the F4 fraction, but a higher rise in the organic (F3) fraction. The co-pyrolyzed biochar illustrated an advantage over the sole use of MSS biochar, signifying the beneficial impact of co-pyrolysis. Similar stability trends were evident in co-pyrolysis with cotton stalk compared to bamboo sawdust. The highest stability was observed in the co-pyrolysis of Pb at 650 °C, registering an I_r_ of 0.93, and Zn from MSS biochar alone at 400 °C, with an I_r_ of 0.31 [28]

At a temperature of 650 °C, both the biochar from MSS pyrolysis and that from co-pyrolysis showed no exchangeable fraction and no reducible fraction for Cd, Cr, Cu and Pb, while only a minimal trace was observed for Zn. The stability of Cr was measured to be I_r_ = 0.93 in both pyrolysis and co-pyrolysis biochar at 650 °C, indicating considerable strength. At 450 °C, Zn exhibited the lowest stability among the elements, with a value of I_r_ = 0.50 for both pyrolysis and co-pyrolysis. In addition, an increase in the residual fraction (F4) was observed in MSS biochar and co-pyrolyzed biochar with zeolite [44].

The results show that Zn exhibits minimal stability in MSS biochar, especially at lower pyrolysis temperatures between 400 and 450 °C. This indicates that the Zn increasingly separates into the F1 and F2 fractions at these temperatures, in contrast to the F3 and F4 fractions.

Nevertheless, the influence of the co-pyrolysis of MSS on the distribution of HMs in biochar is significant, which is due to its ability to convert HMs from the less stable fraction to more stable compounds. This process results in lower direct toxicity and bioavailability compared to biochar derived solely from MSS. It is recommended that future research efforts prioritize the comprehensive analysis of the HM distribution in the co-pyrolysis of MSS with different co-substrates, with particular attention paid to the most prevalent species.

### 3.2. PAHs in MSS Biochar

Polycyclic aromatic hydrocarbons (PAHs) represent a class of semi-volatile compounds characterized by two or more aromatic rings. In biochar, PAHs stand as the most prevalent contaminants among organic pollutants [28]. The pyrolysis process leads to the formation of PAHs through a complex reaction mechanism involving the carbonization and aromatization of organic matter. The bonding of hydrocarbon radicals contributes to the production of heavier aromatic molecules [45]. The concentration of free radicals (FRs) plays a significant role in forming PAHs, and the presence of oxygen catalyzes their creation [46]. Consequently, it is imperative to thoroughly consider these factors, as biochar may encounter oxygen during the cooling or storage phase of biochar.

The correlation between the temperature of MSS pyrolysis and PAH formation during the pyrolysis process remains ambiguous, lacking a definitive trend. Nevertheless, research on MSS biochar conducted by Luo et al. and Chen et al. has indicated that PAH levels peak between 400 and 500 °C and decrease with an increasing temperature (>500 °C) [47,48]. Previous studies indicate that the bioavailable fraction (called the freely dissolved concentration, C_free_) of PAHs in MSS biochar generally remains below 200 ng/L [49,50,51]. A study by Hale et al. observed that higher pyrolysis temperatures reduce the concentration of bioavailable PAHs, albeit with some exceptions [50]. Consequently, as the temperature rises, the biochar surface becomes increasingly aromatic, thereby attracting PAHs and decreasing their bioavailability due to hydrophobic and π–π electron–donor-acceptor interactions (this suggests that lower temperatures promote PAH formation, while temperatures surpassing 500 °C facilitate the volatilization of formed PAHs).

The International Biochar Initiative (IBI) and the European Biochar Certificate (EBC) have established precise standards for permissible levels of PAHs in biochar. According to the IBI guidelines [52], the acceptable quantity for Σ16 PAHs, as stipulated by the US Environmental Protection Agency (EPA), in biochar should be <300 mg/kg. Biochar with a PAH content exceeding 300 mg/kg is not recommended for use as a soil amendment, according to the IBI’s guidelines [52]. The recommendations by the EBC [53] are characterized by a high degree of rigor and comprehensiveness, covering both premium and basic grades of biochar. In line with these recommendations, a biochar sample is classified as a premium grade if its PAH content is below 4 mg/kg. Conversely, basic grade biochar is defined as such when it contains a PAH content of up to 12 mg/kg. These recommendations provide valuable guidance for evaluating and comparing different grades of biochar. Legislative regulations on the bioavailable fraction of PAHs in biochar are currently absent, and limited data are available on this aspect [45,50].

Studies have shown that the MSS co-pyrolysis process reduces the PAH concentration in biochar compared to MSS biochar alone [10,54,55]. The research conducted by Kończak et al. revealed that the pyrolysis of MSS at 500 °C with CO_2_ as the carrier gas resulted in a total PAH content of 2263 mg/kg, with 44 ng/L being bioavailable [56]. However, co-pyrolysis of MSS with willow (fresh shoot) at a 6:4 (MSS/willow wt.%) ratio under the same conditions reduced the total PAHs to 797 mg/kg, with 25 ng/L of bioavailable PAHs. Similarly, at 600 °C and 700 °C, pyrolysis of MSS alone yielded total PAHs of 1730 mg/kg and 1449 mg/kg, with bioavailability of 51 ng/L and 46 ng/L, respectively. Co-pyrolysis at these temperatures resulted in total PAHs of 818 mg/kg and 938 mg/kg, with bioavailability of 24 ng/L for both temperatures. Furthermore, the predominant PAHs in MSS biochar alone at 500 °C, 600 °C, and 700 °C were three-ring (phenanthrene, anthracene and naphthalene), while co-pyrolysis biochar exhibited predominant two-ring PAHs. This reduction is attributed to the dilution effect and catalytic cracking. The inclusion of lignocellulosic biomass, such as agricultural residues, in co-pyrolysis dilutes the MSS feedstock, leading to an overall decrease in the PAH concentration compared to MSS alone. Moreover, the addition of biomass feedstocks with higher lignin and cellulose content promotes the formation of more stable aromatic structures (phenolic compounds) rather than PAHs during pyrolysis [35,57]. Furthermore, a higher pyrolysis temperature (>700 °C) during MSS co-pyrolysis supports the cracking and decomposition of PAHs, thereby reducing their concentration in the resulting biochar [57,58]. Consequently, it can be inferred that MSS co-pyrolysis with a suitable co-substrate and high temperature can significantly mitigate PAH levels and their bioavailability in MSS co-pyrolyzed biochar.

However, limited evidence exists to indicate the impact of PAHs (total or bioavailable) on biochar’s toxicity. Additionally, comprehensive investigations regarding biochar aging and PAH bioavailability are scarce. Therefore, further research must prioritize monitoring and regulating PAH bioavailability in biochar, particularly those originating from MSS, given its current utilization in co-pyrolysis.

### 3.3. PCDD/Fs in MSS Biochar

PCDD/Fs (persistent environmental pollutants commonly referred to as “dioxins”) are characterized by a triple-ring structure comprising two benzene rings linked by one or two oxygen atoms. Recent research indicates that the PCDD/Fs level in biochar is typically lower compared to HMs, PAHs and PFASs [59]. The MSS generally contains a low level of chlorine, approximately 0.05–0.18% on a dry matter basis [48,60]. However, if a raw material has a high chlorine content (over 1%), the pyrolysis process can lead to the formation of PCDD/Fs [61]. This underlines the crucial importance of careful selection of the co-substrate during the co-pyrolysis process with MSS.

The formation of PCDD/Fs involves two distinct pathways: the homogeneous (gas phase) route and the heterogeneous (biochar particle surface) route [62]. During pyrolysis, the homogeneous route occurs within a temperature range of approximately 500–800 °C [62]. This pathway comprises four main pathways, including the cyclization of polychlorobiphenyls, polychlorobiphenyl ethers, the chlorination of dibenzofuran and the dichlorination of PCDF. During this process, HCl is released into the gas phase, producing highly chlorinated compounds such as polychlorophenols, polychlorobenzenes or PCBs [62,63,64]. These compounds serve as precursors for the formation of PCDD/Fs by polycondensation catalyzed by metals [63,65].

The heterogeneous route runs in a temperature range of 200–450 °C and has two different formation paths [62,63,64]. These pathways include chloroaromatic and de novo reactions. The formation process depends on the amount of O, and the reaction rate depends on the rate of C decomposition [64,66]. HMs, especially Cu, serve as catalysts for the oxidation of C, chlorination and dichlorination of organic products and act as a channel for chlorine transfer between the gas and solid phases [64,67].

Research on the determination of PCDD/Fs in biochar from MSS is scarce; however, the existing results indicate that higher pyrolysis temperatures generally lead to lower PCDD/Fs levels. Increasing the temperature from 300 to 700 °C can significantly reduce the PCDD/Fs concentration in biochar [58,68].

A study by Sørmo et al. demonstrated that MSS biochar, produced within the temperature range of 500 to 800 °C, exhibits an exceptional PCDD/Fs removal efficiency exceeding 99.9% [69]. Furthermore, a comparison between MSS biochar and raw MSS showed that under the corresponding pyrolysis conditions (500–700 °C), 98–99% of the PCDD/Fs and about 90% of the PCDD/Fs toxicity quantified in the I-TEQ (International Toxicity Equivalent Quantity) were effectively eliminated [70]. It is noteworthy that the IBI regulation has set 0.017 ng/g d.m. as the default limit for PCDD/Fs in biochar. In comparison, the EBC Regulation [53] sets the standard at 0.02 ng/g d.m. based on the biochar classes (AgroBio Class II and Agro Class III) [71].

### 3.4. PFASs (PFOS/PFOA) in MSS Biochar

Perfluorooctane sulfonate (PFOS) and perfluorooctanoic acid (PFOA) are two of the most widely used and hazardous per- and polyfluoroalkyl substances (PFASs), often referred to as “Forever Chemicals” They belong to a class of highly fluorinated aliphatic compounds characterized by at least one fully fluorinated methyl or methylene carbon atom and either a straight or branched alkyl chain [72,73]. Previous research has shown that the pyrolysis process effectively eliminates PFAS compounds. For example, Kundu et al. [74] reported that over 90% of PFOAS and PFOA in MSS were eliminated during pyrolysis of MSS biochar. In contrast, the resulting biochar retained its ability to absorb pre-existing PFASs contamination. Additionally, Kim et al. [75] found that the concentration of PFOA and PFOS in MSS biochar was between 10.6 and 11.5 ng/g and 4.8 and 6.3 ng/g, respectively. However, the total amounts of PFOA and PFOS in the biochar decreased by up to 50% when the total weight loss of the biochar during pyrolysis was taken into account.

Notably, the existing research lacks studies focusing on PFOS/PFOA in MSS co-pyrolysis biochar, with the few available studies focusing mainly on MSS biochar alone. This lack of information on PFASs in MSS biochar and co-pyrolysis biochar makes it difficult to determine the key factors that will ensure a reduction or complete removal of PFASs in the resulting biochar. Consequently, there is a clear need for studies that focus on the performance of MSS co-pyrolysis biochar with respect to PFASs, as well as further research on the range of biochar produced at different pyrolysis temperatures to determine the factors that influence the sorption of PFASs.

### 3.5. Other Contaminants in MSS Biochar

Apart from the conventional pollutants mentioned above that may be present in biochar, there are some studies that show that biochar may contain other potentially hazardous compounds depending on its source material. One example is free radicals (FRs), which can potentially contribute to the toxicity of biochar. Although studies on the determination of free radicals in biochar from MSS and MSS co-pyrolysis are very limited, the extension of studies to other feedstocks suggests that chemisorption and electron transfer are the mechanisms for free radical formation. FRs generate oxygen species (ROS) that can damage DNA in cell structures [76].

Studies have shown that microplastics (MPs) in raw MSS vary in size from less than 1 μm to more than 5 mm, with fibers and fragments being the most common shapes observed [77,78,79]. The two most common types of MPs in MSS, polyethylene (PE) and polypropylene (PP), are completely degraded when the pyrolysis temperature reaches 450 °C [80,81]. At 500 °C, the total MPs concentrations are reduced from hundreds of particles per gram to 1–2 particles per gram, with no MPs in the 10–50 μm size range remaining [80]. Thus, the thermal decomposition of PE and PP shows a dramatic mass loss between 400 and 500 °C, with complete degradation above 500 °C, leaving no noticeable residue. Similarly, polyethylene (PET) starts decomposing above 450 °C and transitions to the gas phase within minutes at temperatures overs 500 °C [80]. In spite of MP being a pollutant, recent studies have shown that the presence of MPs in MSS co-pyrolysis enhances the carbon structure, pore structure and surface properties of biochar when used [30]. Similarly, Mujtaba et al. showed that biochar from MSS co-pyrolyzed with MPs has a potential to be used in soil remediation [82].

Thus, it is important to recognize the possible presence of additional pollutants in the MSS biochar that could affect its suitability for soil amendment. In particular, the presence of MP, despite its status as an impurity, shows positive effects on the biochar properties when subjected to co-pyrolysis with MS biochar. Consequently, the enrichment of MSS with MP has the potential to improve the physical and chemical properties of the resulting biochar and thus significantly increase its overall quality.

Ultimately, co-pyrolysis represents a sustainable and environmentally conscious approach to reducing the pollutants in MSS biochar. This reduction can be attributed to several mechanisms. One key mechanism is the dilution effect, where the concentration of pollutants decreases due to the presence of two or more substrates in the co-pyrolysis process. This effect helps stabilize the HMs present in the MSS during co-pyrolysis [2]. Additionally, synergistic chemical interactions between MSS and its co-substrate enhance the pollutant stabilization. The introduction of a co-substrate promotes the formation of stable metal complexes, reducing the mobility and bioavailability of pollutants, particularly HMs, in the biochar [2]. Furthermore, the catalytic effects from the interactions between MSS and the co-substrate can accelerate the conversion of pollutants into less toxic forms. For instance, HMs in the feedstock may undergo chemical transformations that lead to their stabilization in the co-pyrolyzed biochar, thereby reducing their bioavailability and toxicity [2]. Co-pyrolysis also optimizes the thermal decomposition process by providing additional carbon and hydrogen, which promotes the degradation of complex organic pollutants and minimizes the formation of toxic compounds. The resulting highly porous structure with a relatively high surface area due to co-pyrolysis improves the adsorption and immobilization of pollutants. The microporous and mesoporous nature of co-pyrolyzed MSS biochar effectively traps and immobilizes pollutants, preventing their leaching into the environment [83].

Regarding organic pollutants, co-pyrolysis has been effective in reducing their concentrations. For example, high-temperature co-pyrolysis (>600 °C) significantly lowers PCDD/F concentrations, with studies showing that more than 99.8% of PCDD/Fs are removed from the feedstock under these conditions [69,84]. Similarly, high-temperature pyrolysis (above 600 °C) has shown promising results in reducing PFAS concentrations, potentially to levels below the detection limit [84]. The impact of co-pyrolysis on PAHs is more complex, although it still results in a notable reduction of the PAH concentrations in co-pyrolyzed biochar compared to pyrolysis alone. It is clear that co-pyrolysis effectively lowers the overall concentration of PAHs and reduces the bioavailability of these pollutants. Moreover, co-pyrolysis influences the aromatic structure of the biochar, shifting toward a predominantly two-ring structure as opposed to the three-ring structure observed with pyrolysis alone [56,69].

However, it is important to note that while the addition of a co-substrate can dilute the pollutant concentrations, this effect is secondary to the primary mechanisms of adsorption, decomposition, and chemical transformation. The co-substrate plays a crucial role in balancing the moisture content and providing additional carbon, which promotes the formation of a stable biochar structure capable of immobilizing pollutants.

## 4. Application of MSS Pyrolysis/Co-Pyrolysis Biochar in Remediation of HM-Contaminated Soils

In recent studies, biochar has been extensively investigated for its potential in environmental remediation, with various preparation and modification techniques being explored to enhance its properties [85]. The use of biochar provides several agronomic benefits to the soil, including increased soil porosity, improved water-holding capacity (WHC), improved aeration, increased stability of soil aggregates, and reduced bulk density and tensile strength [86]. Biochar from MSS pyrolysis and co-pyrolysis is a sustainable approach to soil management and remediation. It holds promising potential for improving long-term agricultural productivity and resilience by recovering nutrients from a critical waste stream and promoting effective resource management. Previous studies have highlighted the benefits of using MSS pyrolysis/co-pyrolysis biochar as a soil amendment, demonstrating its effectiveness in improving soil properties and providing a slow-release nutrient source, contributing to sustainable agricultural practices. MSS biochar also provides active sites for the complexation of HMs and alkalinity for HM precipitation, making it an efficient amendment for HM immobilization [87].

Grutzmacher et al., for example, have shown in a microcosm study that soil amended with MSS biochar reduces the N_2_O emissions caused by fertilizers by 87% [88]. Gonzaga et al. reported an increase in the biomass yield of Indian mustard with tropical soil amended with MSS biochar [89]. Similarly, a 100% increase in the biomass yield of *Paa pretensis* was observed under laboratory conditions when the soil was amended with MSS biochar compared to non-amended soil [90]. In addition, MSS biochar produced at 300 and 500 °C was found to promote the colonization of maize roots with arbuscular mycorrhizal fungi (AMF), which play a crucial role in the production of plant growth hormones, increased nutrient availability, inhibition of root pathogens, and improved mutualistic symbiotic association of maize and AMF [91].

On the other hand, the application of MSS biochar to the soil can have potentially harmful effects on plants, soil invertebrates, microbes and the environment in general. Godlewska et al. and Kavitha et al. provided comprehensive reviews of previous studies confirming the negative effects of MSS biochar [92,93]. For example, due to the presence of various pollutants and large amounts of N and P, MSS biochar can easily lead to eutrophication and soil contamination, which in turn threatens the safety of flora and fauna [94,95]. In addition, the study by Mierzwa-Hersztek et al. found that MSS biochar had a toxic effect on the soil toxicity to *Vibrio fischeri*. It caused luminescence inhibition of 31% to 50% compared to the control soil [90].

Therefore, considerable efforts are currently being made to optimize the conditions of MSS pyrolysis and to modify the MSS biochar in such a way that potential environmental risks are minimized. Consequently, there is great interest in the application of MSS biochar in soil remediation, especially in former and current industrial areas. The potential for such applications is particularly promising for modified MSS biochar, including co-pyrolysis. This section presents the current status of the application of MSS biochar from pyrolysis and co-pyrolysis in the remediation of HM-contaminated soils.

### 4.1. Framework Conditions for the Remediation of HM-Contaminated Soils with MSS Pyrolysis/Co-Pyrolysis Biochar

The use of biochar derived exclusively from MSS pyrolysis for the remediation of HM-contaminated soils has been documented. However, the use of biochar from the co-pyrolysis of MSS for soil remediation has only been researched to a limited extent. Table 2 provides a comprehensive assessment of the different experimental conditions for HM immobilization when using MSS pyrolysis and co-pyrolysis biochar in soil, the types of contaminated soils and the types of HM contamination, as well as the range of soil indicators analyzed, and the methods used to assess HM immobilization. The experiments focused primarily on real contaminated soils, which contained several HMs and came from different locations, such as mining and smelting areas, landfills, fallow land and agricultural areas. Laboratory-scale experimental setups such as pots, greenhouses or columns were used, with the occasional use of indicator plants such as maize, cherry tomatoes, grasses and halophytes to assess HM immobilization.

#### 4.1.1. Temperature

The efficiency of HM immobilization in soil is significantly influenced by both the ambient temperature during the experiment and the pyrolysis temperature of the MSS pyrolysis/co-pyrolysis biochar. Studies conducted by Fang et al. indicated that higher ambient temperatures, specifically 45 °C, promoted the immobilization of Pb in contaminated soil [96]. However, even minor ambient temperature fluctuations had a discernible impact on Pb immobilization. For instance, a decrease in the ambient temperature from 45 °C to 4 °C resulted in an increase in the Pb level from 2.50 to 2.72 mg/L, signifying the re-mobilization of previously immobilized Pb at 45 °C when the temperature decreased to 4 °C. However, the overall effect was not spectacular. Similarly, Hu et al. highlighted the influence of the ambient temperature on the migration and distribution of HMs in soil [97]. Their findings revealed that at 45 °C, the percentage of soil water-soluble Cu doubled compared to 15 °C, underscoring the temperature’s effect on the HM distribution. Moreover, studies by Hasanzad et al. emphasized the significance of the ambient temperature in HM immobilization in soil [98]. The study observed that the ambient temperature could impact the stability of HMs in the soil, as higher ambient temperatures lead to heat loss, causing the movement of cations to available empty spaces due to the loss of water and hydration around them.

Regarding the pyrolysis temperature of MSS pyrolysis/co-pyrolysis biochar, the effects of different MSS biochar pyrolyzed at 300 °C and 650 °C are shown in Table 2. It is evident that the higher pyrolysis temperature of MSS pyrolysis/co-pyrolysis biochar increases the formation of stable fractions on soil amendment. For instance, the investigation by Wang et al. on the effects of different biochars pyrolyzed at 300 °C and 500 °C on soils polluted with Cd and Pb showed that soil amended with MSS biochar pyrolyzed at 500 °C showed a significant increase in the fraction of mobile Cd and Pb converted to the stable fraction compared to soil amended with biochar pyrolyzed at 300 °C [99]. This was demonstrated by DTPA extraction and underlines the central role of temperature in the remediation of HMs in soil. Similarly, the highest Pb and Zn immobilization in soil occurred at the maximum pyrolysis temperature of MSS biochar using AB-DTPA extraction, as shown in the study by Fathianpour et al. on Pb and Zn stabilization in soil by MSS biochar prepared at different temperatures (300 °C, 400 °C, 500 °C) [100].

Table 2 shows a range of test ambient temperatures from 4 °C to 45 °C, with the typical range being between 20 °C and 25 °C and the pyrolysis temperature between 300 °C and 650 °C. It is worth noting that previous research has not directly addressed the correlation between the experimental ambient temperature and the pyrolysis temperature of MSS pyrolysis/co-pyrolysis biochar in HM-contaminated soil, as most studies have only briefly described the effect. Nevertheless, it can be deduced from the few available studies on MSS pyrolysis/co-pyrolysis biochar that a higher ambient and higher pyrolysis temperature significantly increases the immobilization of HMs in contaminated soils when MSS pyrolysis/co-pyrolysis biochar is used for soil amendment. Future studies should focus directly on developing the relationship between the ambient and pyrolysis biochar temperature and how they specifically affect the HM immobilization in soil.

#### 4.1.2. Biochar Rate

The biochar application rate (i.e., biochar dosage) varied from 1% to 8% in the different studies, as shown in Table 2. While some studies have identified the specific rate required for maximum HM immobilization using MSS biochar, other factors such as the temperature during the experiment and pyrolysis temperature of the MSS pyrolysis/co-pyrolysis biochar, experiment duration and pH can also have a significant impact. For example, in a recent greenhouse study, a gradual increase in the rate of MSS biochar from 1% to 5% (*w*/*w*) resulted in a progressive decrease in the bioavailability (DTPA extractable concentrations) of various HMs (Cd, Cr, Cu, Fe, Ni, Pb, Zn) in soil [101]. Similarly, Wang et al. reported that a biochar rate of 5% increases the immobilization of Cd and Pb in soil [99], as explained in the following section. From this, it can be deduced that a biochar content of 1% to 5% is optimal for the immobilization of HMs in the soil, taking into account other factors such as the temperatures (ambient temperature and pyrolysis) that could influence it.

#### 4.1.3. Experimental Duration

The duration of the experiment (soil incubation) has a significant influence on the immobilization of HMs in soil using MSS pyrolysis/co-pyrolysis biochar. The duration of the trials varied from 30 to 360 days, with 60 days being the most common, as shown in Table 2. Moreover, 30- to 60-day short trials and 90- to 360-day long trials were carried out. A long trial duration can change the properties of the biochar, such as the pH, alkalinity and CEC, and consequently affect its immobilization efficiency [102,103]. However, MSS biochar has shown higher resistance to change over time and/or better immobilization efficiency compared to other biochar feedstocks such as bamboo sawdust [103,104]. Therefore, a longer experiment duration is expected to yield more favorable results due to the positive impact of aging [103,104]. For example, a 360-day period of experiment demonstrated that the application of MSS biochar produced at 400 °C reduced the mobility of HMs such as Cr, Cu, Mn, and Zn in contaminated soil. This reduction was attributed to the substantial decreases in the acid-soluble fractions of these HMs by 72.20%, 50.43%, 70.38%, and 29.78%, respectively [105]. Additionally, the 100-day study by Fang et al. demonstrated that a long experimental time and higher temperature favored As immobilization, which was consistent with Pb and Cr immobilization. Hence, ensuring a longer experimental time, possibly >60-day, is key to adequate HM immobilization [96].

#### 4.1.4. Soil Moisture

In HM remediation, the moisture content of the soil (water-holding capacity/field capacity) exerts a considerable influence. It is evident that the moisture content of the soil has a direct influence on the mobility and potential leaching of HMs in remediated soils. Studies dealing with the implementation of MSS pyrolysis/co-pyrolysis have consistently reported a standard soil moisture content of 60% of WHC [6,100,105,106,107,108]. In contrast, Chen et al. [12] have documented that a moisture content of 25% and 35% when using MSS biochar (500 °C) in contaminated soils provides optimal results in terms of the immobilization of HMs (Pb and Cr). Nevertheless, it is noteworthy that maintaining the moisture content of the soil at this level throughout the experimental duration of each study highlights its role in maximizing the immobilization of HMs.

#### 4.1.5. Soil pH

The biochar resulting from MSS pyrolysis/co-pyrolysis has been observed to elevate the soil pH from weakly acidic to slightly alkaline. This effect is attributed to the presence of soluble carbonates and surface functional groups in the biochar [6]. In a study conducted by Fang et al., the pH value was found to increase from 4.0 to 4.3, irrespective of the MSS biochar rate (1–5%) added to the soil [96]. However, when comparing soil amended with MSS pyrolysis biochar and co-pyrolysis biochar (MSS/cotton stalk), it was determined that the latter significantly raises the soil pH compared to MSS biochar alone [108]. Specifically, the soil pH increased by 0.5 units in soil amended with MSS co-pyrolyzed biochar with cotton stalk, in contrast to 0.2 pH units in soil with MSS biochar alone [108]. This elevated soil pH in co-pyrolyzed MSS biochar-amended soil is likely conducive to the immobilization of HMs. A higher soil pH can enhance the effectiveness of HM stabilization in soil through the formation of oxides and hydroxide precipitates [109]. Nevertheless, the specific increase in the soil pH due to co-pyrolyzed MSS biochar varies depending on the temperature of the co-pyrolyzed biochar and the type of co-substrate.

#### 4.1.6. Cation Exchange Capacity (CEC) and Total Organic Carbon (TOC) of Soil

Regarding the increase in the CEC value of the soil, it should be noted that the MSS biochar effectively increases the CEC value simply due to its higher ash content compared to the biochar produced by co-pyrolysis. The CEC value of the soil enriched with MSS biochar was 7.8 cmol/kg, exceeding the CEC value of the soil enriched with co-pyrolyzed MSS biochar (MSS/cotton stalk) of 4.3 cmol/kg [108]. Due to the ion exchange processes and electrostatic attraction forces, a higher CEC improves the adsorption and immobilization of HM cations [110]. The CEC of the soil often correlates with the pH, since as the pH of the soil increases, the deprotonation of the functional groups of the organic matter increases the negative charges on the soil particles, which in turn increases the CEC [111]. In this case, due to the increased CEC, more negatively charged binding sites are available to absorb and immobilize cationic HM species. As a result, the cationic HMs become less soluble, causing the equilibrium to change in favor of their precipitation as insoluble hydroxides, carbonates or oxides [111,112].

Nevertheless, the TOC content of the soil enriched with co-pyrolyzed MSS biochar (MSS/cotton stalk) was higher than that of MSS biochar alone. This is due to the more porous structure of the co-pyrolyzed biochar. This increase in the TOC content significantly improved the efficiency of immobilization of HMs, especially Pb, as it promoted the formation of more Pb complexes with organic ligands in the soil [108].

It is noteworthy that the influence of these conditions on the immobilization of HMs by MSS pyrolysis/co-pyrolysis requires further investigation to sufficiently understand the immobilization mechanism. Further research should aim at a comprehensive understanding of the general conditions affecting HM immobilization to determine the full spectrum of requirements for maximum HM immobilization. In addition, greater attention should be paid to the investigation of MSS co-pyrolysis biochar, as only a limited number of studies have been conducted to date. Biochar from MSS co-pyrolysis represents a new strategy for the effective remediation of HM-contaminated soils.

### 4.2. Effect of MSS Pyrolysis/Co-Pyrolysis Biochar Application on HM Immobilization in Soil

Understanding the fate and behavior of MSS biochar from pyrolysis or co-pyrolysis in relation to the mobility of HMs in soil is a crucial factor for its practical application. Previous studies have highlighted the benefits of biochar from different feedstocks in terms of HM immobilization. However, to assess the environmental impact of the suitability and optimal dosage of MSS biochar from pyrolysis or co-pyrolysis in soil, it is important to understand the mechanism of HM immobilization in soil when amended with biochar.

The evaluation of HM immobilization in soil can be performed by several methods, such as the toxicity-leaching procedure (TCLP), simple bio-accessibility extraction test (SBET), or sequential extraction procedures such as the Tessier sequence extraction procedure (TSEP) and the European Community Bureau of Reference (BCR) sequential extraction procedure [113,114,115,116]. The TCLP is efficient in extracting the most active part of HMs and evaluating the risk of the tested soils [116,117,118,119]. The SBET simulates the human digestive system and evaluates the risk changes to humans during the immobilization process, revealing the potential risk of chemical stability [116,117]. The TSEP divides HMs in soils into five different fractions, while the BCR divides into four fractions. Both sequential extraction protocols can help determine the mechanism of HM immobilization in soil amended with different amendments, including biochar [116,117,118,120].

**Table 2:** Conditions for HM immobilization and remediation in soil with MSS biochar from pyrolysis/co-pyrolysis.

Substrate Type/Temperature of Pyrolysis	Soil Type/Contamination Source/ Location	Crop Type	Type of HM	Experimental Conditions				Soil Indicators	Assessment of HM Immobilization	Ref.
				Scale, Type of Experiment	Biochar Rate	Time of Experiment (Days)	Soil Moisture			
**Pyrolysis**										
MSS from mechanical-biological with/without tertiary treatment and with/without sludge stabilization/600–650 °C	Real contaminated soils/Zn-rich, smelter, As-rich, brownfield, garden/Czech Republic	Absent	As, Cd, Cu, Pb, Sb, Zn	Laboratory pots (150 mL soil and 4.5 mL MSS biochar)	3% (*v*/*v*)	30	70% of WHC	Analyses in pore water: pH, Eh, EC, anion, total carbon, DOC, major and trace elements	Exchangeable fraction for Zn, Pb, Cd, Cu with 0.11 M acetic acid, exchangeable fraction Sb and As with ammonium sulphate	[121]
Dewatered SS/500 °C	Real contaminated soil/reservoir area/China	Absent	Cr, Pb	Laboratory polymethyl methacrylate columns	nr	35	25% or 35%	pH	HM fractionation with TSEP	[12]
Anaerobically digested, dewatered MSS/600 °C	Natural infertile oxisol soil (silty clay)/USA	Maize (*Zea mays* L., cv. Super Sweet #9)	As, Cd, Co, Cr, Ni, Pb, Se	Greenhouse bioassays (2.1 kg of soil)	2.5% (*w*/*w*)	35	50% (*w*/*w*)	Soil pH, TOC, TN, P, extractable basic cations (Ca, Mg), biomass yield	HM accumulation in above ground of maize biomass	[13]
MSS/550 °C	Chromosol/recycled organic agriculture site/Australia	Cherry tomato (*Lycopersicon esculentum*)	Ag, As, Ba, Be, Bo, Cd, Cr, Co, Cu, Ni, Pb, Sb, Sn, Se, Sr, Zn,	Glasshouse pots (6 kg soil) at 20–26 °C	10 t/ha	112	nr	EC, pH, TN, extractable P, CEC, exchangeable cations, micronutrients	Accumulation of HMs in fruit by ICP according to the US EPA 6010	[14]
MSS/400 °C	Real contaminated soil/mining site, battery manufacturing waste site, electroplating site, and industrial waste disposal site/China	Absent	As, Cd, Cr, Ni, Pb	Laboratory glass dishes, at different soil temperatures (4, 25, 45 °C)	1–5% based on dry mass	100	50	pH	HM stability by acid batch extraction. HM fractionation with sequential extraction	[96]
MSS/300 °C, 400 °C, 500 °C	Real contaminated soil/mining site/Iran	Absent	Pb, Zn	Laboratory plastic containers (40 g of soil)	3% (*w*/*w*)	60	60% of WHC	pH, T	Estimation of bioavailable fraction with ammonium bicarbonate-diethylene triamine pentaacetic acid (AB-DTPA) method	[100]
MSS/500 °C	Haplic cambisol (sandy-loam)/mediterranean landscape/Spain	Absent	Cd, Cu, Ni, Pb, Zn	Laboratory glass vessels (100 g of soil) at 28 °C	4% and 8%	200	60%	TMC, soil perspiration (cumulative CO_2_ evolution)	Estimation of bioavailable fraction with diethylenetriaminepentaacetic acid-CaCl_2_-triethanolamine (DTPA)	[106]
MSS/400 °C	Spiked Alumi-Plinthic Acrisols/agricultural site/China	Absent	Cr, Cu, Mn, Zn	Laboratory pots (100 g of soil)	5% (*w*/*w*)	360	60% of WHC	pH	HM fractionation with BCR sequential extraction	[105]
MSS/500 °C	Real contaminated soil/mine site (acidic and basic)/Spain	*Sarcocornia fruticosa* (L.)	Cd, Pb, Zn	Laboratory PVC columns	6% based on dried weight	303	nr	pH, Eh, T, EC, WSOC evolution	HM fractionation with BCR sequential extraction	[104]
MSS/650 °C	Real contaminated soil/composite soil/China	Maize (*Zea mays* L.)	Cr, Ni, Pb	Laboratory pots (1 kg of soil)	2% (*w*/*w*)	90	60–70% FC	nr	Modified sequential extraction procedure (8-steps). Acid digestion method used to measure HM in plants parts	[107]
MSS/300 °C, 500 °C	Real contaminated soil/paddy soil/rice field/China	Absent	Cd, Pb	Laboratory containers (600 g of soil) at 25 °C	1%, 3%, 5%	60	70%	nr	Estimation of bioavailable fraction with DTPA and HM fractionation with BCR sequential extraction	[99]
**Co-pyrolysis**										
MSS + Rice straw/400 °C	Real contaminated soil/paddy soil (clay)/China	Absent	Cd	Greenhouse plastic pots (500 g of soil) at 25 °C	3% (*w*/*w*)	60	60%	pH, CEC, SOC, AP, chemical speciation of Cd	HM fractionation with TSEP, estimation of bioavailable fraction with 0.1 mol/L CaCl_2_ solution	[6]
MSS + Cotton stalks/650 °C	Real contaminated soil/sandy loam/farm near a Pb–Zn mining site/China	Ryegrass	Cu, Pb, Zn	Laboratory pots at 18–23 °C	7.5 t/ha	60	60% of WHC	pH, EC, CEC, AP, AK, AN, TOC	Estimation of fraction with DTPA and CaCl_2_ and mobility, HM fractionation with BCR sequential extraction	[108]

AK—available potassium, AN—available nitrogen, AP—available phosphorus, CEC—cation exchange capacity, EC—electrical conductivity, Eh—soil redox potential, FC—field capacity, nr—not recorded, SOC—soluble organic carbon, TMC—total mineralization co-efficient, TOC—total organic carbon, WHC—water-holding capacity, WSOC—water soluble organic carbon.

#### 4.2.1. MSS Biochar from Pyrolysis

MSS biochar is widely acknowledged as a versatile material with the potential to enhance soil quality, remediate contaminants, and manage excess MSS generated in WWTP [122,123]. The study by Wang et al. on Cd- and Pb-contaminated field paddy soil highlighted that MSS biochar at a rate of 5% exhibited optimal performance for immobilization of Cd and Pb in soil during a 60 d incubation [99]. Moreover, the immobilization efficiency for HMs was better for MSS biochar produced at 500 °C than 300 °C. The Cd bioavailability (based on DTPA extraction) under the optimum MSS biochar rate was reduced from 47.45 to 12.82 mg/kg, while for Pb from 90.23 to 15.91 mg/kg. A high reduction in the HM bioavailability was related to the high SSA of biochar, which facilitated HM adsorption. The presence of MSS biochar confirmed the efficient Cd and Pb redistribution in amended soil. The results of the BCR extraction showed that at higher pyrolysis temperature and application rate, more of the mobile fraction (acid soluble) of Cd and Pb was converted to the stable fraction (residual). For example, the share of Cd in mobile acid-soluble fraction decreased by ca. 50% (from 25.13 mg/kg to 12.64 mg/kg), while in the most stable residual fraction, it increased by 94% (from 12.98 mg/kg to 25.20 mg/kg). Similarly, there was a 73.07% decrease (from 27.37 mg/kg to 7.37 mg/kg) in the acid-soluble fraction of Pb, while the content of the residual fraction (F4) increased by 93.21% (from 27.67 mg/kg to 53.46 mg/kg).

Additionally, Fang et al. investigated the immobilization of anionic As and Cr and cationic Cd, Ni and Pb in soils differing in terms of the pollution source, i.e., mining site, battery-manufacturing site, electroplating site and industrial disposal site [96]. The addition of MSS biochar improved the immobilization of anionic and cationic HMs, which was enhanced by increased the ambient temperatures and extending the incubation periods. The authors stated that the method of applying MSS biochar significantly influenced its effectiveness for HM immobilization. Uniform mixing of the biochar with the soil was more effective in delaying HM breakthrough and reducing the amount of HMs released than when using it as a separate layer. The MSS biochar caused a change in the oxidation state of the HMs, which can alter their bioavailability and toxicity. The amendment not only absorbed Cr(VI) but also facilitated its partial reduction to the less toxic Cr(III). Similarly, immobilized As(III) was oxidized to As(V). While MSS biochar has potential for HM stabilization, its effectiveness can be compromised by the presence of other competing HM ions. The immobilization of Pb was related to the direct sorption of liable Pb onto the MSS biochar surface. As a result, Pb was redistributed from exchangeable into reducible and oxidizable fractions. The immobilization process appeared to be robust, largely irreversible, and characterized by strong binding mechanisms. For Zn, the effectiveness of MSS biochar was less pronounced, largely due to competing interactions in the soil matrix, suggesting that targeted strategies are required when multiple HMs are present. Uniform mixing of the MSS biochar in contaminated soils was more effective for the long-term immobilization of Cd and Ni, reduced their release during column leaching, and provided better resilience to environmental fluctuations such as pH changes.

Furthermore, Chen et al. conducted a study to investigate the use of MSS biochar in the passivation and remediation of Cr and Pb contamination in soil obtained from a reservoir area [12]. The study indicated that the addition of MSS biochar to the soil resulted in the reduced bioavailability and mobility of Cr and Pb, leading to their transformation into more stable forms in comparison to the control soil. Specifically, Cr was redistributed from exchangeable fraction (decrease by 73%) into carbonate (increase by 13.2%), reducible (increase by 23.9%) and oxidizable fractions (increase by 30.8%). The redistribution of Pb concerned transformations from the exchangeable and carbonate fraction (decrease by 41.9%) into the reducible fraction (increase by 18.5%). These transformations were facilitated by a suitable soil moisture content, 25% for Pb and 35% for Cr. The combination of the mineralogical composition (presence of silicon dioxide, silicon disulfide, aluminum phosphate), structural features (presence of structured minerals), and chemical functionalities (porosity, alkalinity, hydroxyl and carbonyl groups) in the MSS biochar contributed to the effective immobilization of Pb and Cr. Through complexation, coordination, reduction and precipitation processes, MSS biochar reduced the toxicity and mobility of these HMs, improved the soil quality and reduced the environmental risks. The various mechanisms identified highlight the multifaceted role of MSS biochar in the immobilization of HMs and provide a comprehensive approach to soil remediation.

Additionally, Rashid et al. observed that the application of MSS alone and MSS biochar at 2% effectively reduced the potential mobility of HMs (Cr, Ni, Pb) while increasing the residual fraction in field-contaminated soils [107]. The MSS biochar showed better properties in terms of the surface morphology, i.e., a larger surface area (25.2 m^2^/g vs. 16.2 m^2^/g) and a larger total pore volume (0.10 cm^3^/g vs. 0.07 cm^3^/g). Various minerals, such as quartz, calcite and fluorite, were identified in both supplements, with quartz dominating in the MSS biochar. The MSS biochar was more efficient in increasing the stability of Pb than Cr and Ni (based on the amount of HMs in the residual fraction) compared to MSS alone. Despite the positive effect of HM immobilization, the differences in the HM redistribution with these two amendments were not significant. The reduction of HM mobility by the addition of amendments could be achieved by different mechanisms such as complexation, physiochemical adsorption and ion exchange. Suppression of HMs by the amendments increased maize growth and mass production. In addition, MSS biochar had comparable nutrient retention rates to MSS alone but had a higher concentration of available nutrients (i.e., K, P, N, Ca and Fe), which are important for soil fertility.

MSS biochar has demonstrated effectiveness in remediating contaminated soil by reducing the mobility of HMs and promoting their transformation from the most readily available to the least available fractions through adsorption-based complexes. Given the enhanced properties observed when MSS is co-pyrolyzed with other feedstocks, prioritizing the use of modified biochar (co-pyrolyzed) is crucial to maximize HM immobilization. Therefore, it is imperative to consider MSS co-pyrolysis biochar for optimal soil remediation.

#### 4.2.2. MSS Biochar from Co-Pyrolysis

Current research demonstrates the effectiveness of MSS biochar in remediating HM-contaminated soil, with co-pyrolysis showing potential to further enhance these properties. However, the impact of co-pyrolysis biochar on HM immobilization in soils is not well studied, and the related mechanisms remain largely unexplored.

To date, only two studies have specifically examined the application of MSS co-pyrolysis biochar for HM-contaminated soil remediation. These studies suggest that the mechanisms of HM immobilization in soil amended with MSS co-pyrolyzed biochar are similar to those of MSS biochar alone, involving ion exchange and mineral precipitation. For example, the immobilization of Pb involves ionic exchange and the precipitation of pyromorphite-type minerals [Pb5(PO_4_)3X; X = F, Cl, B, or OH]. The introduction of MSS co-pyrolyzed biochar generally increases the soil pH, facilitating the precipitation of HM hydroxides, carbonates, and phosphates, thus reducing the HM mobility. Also, the formation of pozzolanic reaction products induced by the increased solubility of Al and Si as a result of the high pH facilitates Pb precipitation [124]. Furthermore, biochar surfaces rich in alkali metals such as Ca and Mg readily exchange with positively charged Pb ions, resulting in decreased mobility through cation exchange with Ca and Mg [125]. Additionally, the surfaces of biochars rich in π-electrons interact with Pb deficient in π-electrons, leading to Pb–π electrostatic interaction [125].

Nevertheless the presence of sulfides in the biochar leads to the formation of insoluble metal sulfides, which bind HMs via surface complexation and cation exchange. These effects are enhanced by the high SSA and the functional groups present in the co-pyrolyzed biochar, promoting HM adsorption.

Sun et al. studied the bioavailability of Cd in soil, especially within the first seven days, suggesting that MSS co-pyrolyzed biochar can effectively immobilize Cd in paddy soil in the short term [6]. The study found that the intensity of the -OH peak in the biochar decreased over a 60-day period, suggesting that -OH groups contribute to Cd immobilization through surface complexation. In addition, the decrease in the PO_4_^3−^ and CO_3_^2−^ peaks indicated their involvement in Cd immobilization through surface precipitation. The co-pyrolyzed biochar converted more Cd from the acid-soluble fraction to the oxidizable fraction than the MSS biochar alone, suggesting that complexation plays a more important role in co-pyrolyzed MSS biochar than in MSS pyrolysis alone [108]. The higher immobilization efficiency of co-pyrolyzed MSS biochar was due to the higher pH, CEC and available P content in the soil. In addition, the higher immobilization with MSS biochar was primarily due to the larger SSA, developed pore structure, and functional groups such as π–π* bond, which provided more sites for Cd immobilization and favorably affected the reaction with Cd in the soil. In addition, the high soluble organic C content in the pyrolyzed biochar-amended soil promoted the formation of Cd complexes with organic ligands, which indirectly enhanced the Cd immobilization. The overall Cd immobilization efficiency (based on the reduction of the bioavailable fraction) was 85.61% for co-pyrolyzed MSS biochar and 66.89% for MSS biochar.

Another study by Wang et al. showed that co-pyrolyzed biochar from MSS and cotton stalks is a promising approach for the remediation of HM-contaminated sandy loam soil in terms of reducing the bioavailability of HMs, converting HMs into stable fractions, improving soil properties and promoting plant growth [108]. The co-pyrolyzed MSS biochar reduced the bioavailable forms of Pb, Cu and Zn by 19.0%, 34.9% and 18.2%, respectively, more effectively than MSS biochar. This reduction in bioavailability was also reflected in the lower accumulation of these metals in ryegrass, which decreased by 28.6%, 50.1% and 30.0%, respectively, compared to the SSB amendment. The co-pyrolyzed MSS biochar distributed more HMs from the acid-soluble fraction to the oxidizable fraction than the MSS biochar. This indicates that the complex formation mechanism played a more important role in the co-pyrolyzed MSS biochar and contributed to the long-term immobilization of HMs. The better-developed pore structure and higher SSA value of the co-pyrolyzed MSS biochar provided additional adsorption sites for HMs and improved the immobilization. Various mechanisms were involved in the immobilization of HMs, such as surface adsorption, ion exchange, co-precipitation and complex formation. The presence of functional groups and a higher soil pH facilitated the precipitation of HM hydroxides, carbonates and phosphates, which further reduced the mobility of HMs. The high organic C content in the soil enriched with co-pyrolyzed MSS biochar promoted the formation of complexes with HMs, which improved the immobilization of HMs. In addition, this biochar improved soil properties such as the WHC and pH and provided more essential nutrients (P, N and K). The positive changes in the HM immobilization and soil properties were important for plant growth. Although both biochar types promoted ryegrass growth, the co-pyrolyzed MSS biochar showed a more pronounced effect, increasing the dry biomass of ryegrass by 1.5-fold compared to the control soil, while the MSS biochar increased it by 1.3-fold. Despite the effectiveness of MSS co-pyrolyzed biochar in immobilizing HMs, the study points to the need for long-term assessments to ensure the sustainable immobilization of HMs and to evaluate potential environmental risks associated with the release of HMs from biochar over time.

In summary, the mechanism of HM immobilization through MSS biochar, whether via pyrolysis alone or co-pyrolysis, is a complex process. The interaction between MSS biochar and HMs involves both direct and indirect immobilization mechanisms. Directly, the unique structure of MSS biochar influences HMs’ behavior through chemical and physical interactions as well as precipitation. This is distinct for MSS pyrolysis alone compared to co-pyrolysis. Indirectly, MSS biochar modifies the soil environment, altering its chemical, physical, and biological properties to facilitate pollutant immobilization.

**Figure 4:**
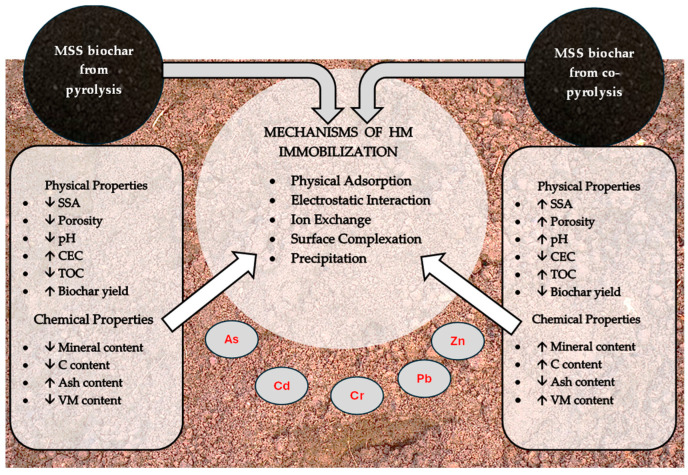
Main mechanisms of HM immobilization in soil with pyrolyzed and co-pyrolyzed MSS biochar (↑ increase; ↓ decrease).

Various mechanisms are at play during these interactions, including cation exchange, precipitation, surface complexation with functional groups, and electrostatic interactions (Figure 4). These mechanisms, however, depend on factors such as the soil type, HM type and concentration, biochar rate, and the aging process of the biochar. For example, Cd is primarily immobilized through cation exchange and precipitation as CdCO_3_, and over time, it is further stabilized by organic functional groups within the biochar matrix [126]. As the biochar ages, mineral dissolution and re-precipitation both reduce its surface area and increase its organic functional groups, enhancing its adsorptive capacity [127].

The behavior of As immobilization with MSS biochar differs significantly from other HMs. While biochar addition can improve the soil conditions for immobilizing cationic heavy metals like Cd and Pb, these same modifications can facilitate the mobilization of anionic metalloids such as As. This is largely due to the electrostatic repulsion and competitive interactions between As and phosphate ions (PO_4_^3−^) for the sorption sites on the biochar [128].

It should also be noted that the interaction of HMs with biochar can trigger various immobilization mechanisms that are influenced by mineral occlusion, sequestration within the biochar pores and the development of π bonds with electron-rich domains on the aromatic groups of the biochar [42]. Comparative assessments indicate that MSS co-pyrolysis biochar can exhibit superior properties for remediating HM-contaminated soil and enhancing soil quality compared to MSS biochar alone (Figure 4). Although research is still limited, MSS co-pyrolysis biochar shows promise and warrants further investigation to fully understand its potential for HM immobilization.

#### 4.2.3. Long-Term Stability of MSS Biochar in Soil

**Figure 5:**
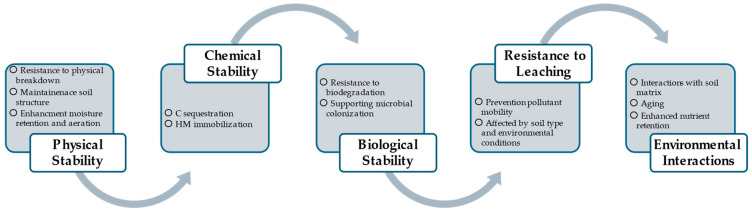
Factors affecting biochar stability in the soil environment.

The stability of MSS biochar in soil is an important factor when considering its use for soil amendment or remediation. Several factors contribute to the overall stability of this biochar and affect its longevity and effectiveness. These factors include the physical, chemical and biological stability of biochar as well as its resistance to leaching and interactions with the soil environment (Figure 5).

As biochar ages, its resilience to physical degradation in soil remains notably high, largely due to its persistent nature [33,35]. Additionally, the enduring impact of MSS biochar on soil aggregates and its contribution to an enhanced soil structure are attributed to its chemical and thermal properties [18]. This results in increased water-holding capacity and improved aeration [129].

The sustainable effectiveness of MSS biochar is further demonstrated by its ability to immobilize certain soil pollutants, thereby reducing their availability and potential for leaching. This immobilization is facilitated by its stable chemical properties, which effectively bind pollutants and limit their release into the environment [33]. Despite the presence of some pollutants in MSS biochar, it can still exert a positive influence on the structure of the microbial community in the soil. By providing a stable habitat and additional nutrients, it promotes increased microbial activity and diversity over extended periods [33].

The myriad benefits associated with the long-term stability of MSS biochar underscore its sustainability as an environmentally friendly technique. The application of MSS biochar in soil not only augments soil nutrient content but also ameliorates soil properties [33,129]. Nevertheless, continued research and field trials are essential to fully understand the effects and refine its use in soil remediation.

## 5. Conclusions and Future Perspectives

This comprehensive review of biochar derived from the pyrolysis and co-pyrolysis of municipal sewage sludge (MSS) highlights its potential to remediate soils contaminated with heavy metals (HMs). The effectiveness of HM immobilization is influenced by several factors, including the pyrolysis temperature, biochar application rate, soil pH and soil moisture content. Studies indicate that MSS biochar produced at higher temperatures (e.g., 500 °C) immobilizes HMs such as Cd and Pb more effectively than at lower temperatures. Co-pyrolysis of MSS with other feedstocks such as cotton stalks, rice straw and fermentation residues from food waste further improves the physical and chemical properties of the resulting biochar, leading to improved immobilization of HMs and soil quality. Co-pyrolyzed biochar has a higher specific surface area (SSA), pH and nutrient content, making it more effective for long-term soil remediation.

However, despite the promising results, there are issues related to the presence of micropollutants such as polycyclic aromatic hydrocarbons (PAHs), per- and polyfluoroalkyl substances (PFAS) and persistent chlorinated organic compounds (PCDD/Fs) in the biochar. The formation and stability of these pollutants requires careful testing to ensure that the use of MSS biochar does not pose any additional environmental risks.

Future research on MSS biochar should focus on optimizing the co-pyrolysis parameters, including the ratio of feedstocks and pyrolysis conditions, to improve the properties of biochar for soil remediation. It is critical to investigate different temperature ranges, heating rates and residence times to determine the most effective conditions for producing high-quality biochar. Investigating the optimal ratio of MSS to different co-substrates is essential for maximizing the benefits of soil remediation.

Moreover, there is a need to expand the immobilization testing of MSS biochar co-pyrolyzed with substrates other than cotton stalks and rice straw to find the most effective combinations for specific soil types and HMs. Detailed studies on the mechanisms of HM immobilization with co-pyrolyzed MSS biochar, including patterns of HM redistribution in soil are crucial.

Long-term studies are essential to assess the effectiveness of HM immobilization and to investigate the potential release of HMs and organic pollutants over time to ensure the sustainable use of biochar for the remediation of HM-contaminated soils. Experiments in plant–soil systems are also important to evaluate the effects of co-pyrolyzed MSS biochar on plant growth, soil health and microbial activity, which are crucial for the restoration of degraded areas.

In addition, detailed studies on the interactions between co-pyrolyzed MSS biochar and soil components, including organic matter, minerals and microbial communities, are needed to understand the long-term stability and effectiveness of HM immobilization. A comprehensive life cycle assessment is also essential to fully understand the environmental impacts of co-pyrolyzed MSS biochar in remediated soils.

All of these activities require interdisciplinary research in collaboration with environmental scientists, chemists, agronomists and engineers to address the challenges associated with the application of MSS biochar and its impact on the soil environment.

## Figures and Tables

**Table 1:** Comprehensive physical and chemical properties of biochar from MSS alone and MSS mixed with various co-substrates.

Feedstock		Temp (°C)		Mixing Ratio	Physical Properties			Proximate Analysis			Ultimate Analysis							Ref.
					SSA (m^2^/g)	TPV (cm^3^/g)	APW (nm)	VM	A	FC	C	H	N	O	S	H/C Molar	O/C Molar	
	**MSS + Bamboo Sawdust (BS)**																	
	MSS	400		1:0 *	5.5	nr	nr	nr	64.2	nr	21.9	1.9	3.1	72.3	0.9	1.0	2.5	[11]
		500		1:0 *	7.7	nr	nr	nr	69.8	nr	21.2	1.2	2.8	73.9	0.8	0.7	2.6	
		600		1:0 *	6.0	nr	nr	nr	74.0	nr	19.9	0.7	2,0	76.5	0.9	0.4	2.9	
	MSS + BS	400		1:1 *	2.8	nr	nr	nr	52.6	nr	31.2	1.8	2.1	64.3	0.5	0.7	1.5	
		500		1:1 *	4.5	nr	nr	nr	58.3	nr	30.4	1.6	2.0	65.4	0.6	0.6	1.6	
		600		1:1 *	8.5	nr	nr	nr	61.9	nr	30.2	1.2	1.8	66.4	0.5	0.5	1.6	
	MSS	1:0 *		5.5	nr	nr	nr	nr	nr	nr	21.9	1.9	3.1	8.1	0.9	1.0	0.3	[22]
		1:0 *		7.1	nr	nr	nr	nr	nr	nr	32.4	0.6	1.7	4.2	1.1	0.2	0.1	
	MSS + BS	400		1:1 **	2.8	nr	nr	nr	nr	nr	31.2	1.8	2.1	11.7	0.5	0.7	0.3	
		700		1:1 **	7.6	nr	nr	nr	nr	nr	32.4	0.9	1.4	0.8	0.6	0.3	0.02	
	MSS	550		1:0 *	31.3	nr	nr	nr	78.4	nr	14.4	0.9	2.0	2.4	2.0	0.7	0.1	[23]
	MSS + BS	550		4:1 *	20.4	nr	nr	nr	70.0	nr	21.1	0.9	1.9	4.3	1.9	0.5	0.2	
	**MSS + Sawdust from Furniture (SD)**																	
	MSS	300		10:0 *	nr	nr	nr	nr	60.7	nr	20.9	7.4	3.5	7.4	nr	4.3	0.3	[24]
		400		10:0 *	nr	nr	nr	nr	65.6	nr	17.7	6.1	2.5	8.2	nr	4.1	0.3	
		500		10:0 *	nr	nr	nr	nr	68.5	nr	17.0	6.0	2.0	6.5	nr	4.3	0.3	
		600		10:0 *	nr	nr	nr	nr	70.4	nr	16.3	6.0	1.7	5.6	nr	4.4	0.3	
	MSS + SD	300		9:1 *	nr	nr	nr	nr	55.2	nr	22.0	7.9	3.3	11.6	nr	4.3	0.4	
		300		7:3 *	nr	nr	nr	nr	46.1	nr	24.3	6.5	3.9	19.2	nr	3.2	0.6	
		300		5:5 *	nr	nr	nr	nr	41.0	nr	26.7	8.01	3.0	21.4	nr	3.6	1.0	
		400		9:1 *	nr	nr	nr	nr	56.9	nr	23.8	7.0	2.5	9.8	nr	3.5	0.3	
		400		7:3 *	nr	nr	nr	nr	47.6	nr	34.8	4.3	2.6	10.7	nr	1.5	0.2	
		400		5:5 *	nr	nr	nr	nr	43.7	nr	43.4	7.6	2.2	3.1	nr	2.1	0.1	
		500		9:1 *	nr	nr	nr	nr	61.9	nr	23.9	4.9	2.3	7.0	nr	2.5	0.2	
		500		7:3 *	nr	nr	nr	nr	50.6	nr	40.6	3.7	2.4	3.7	nr	1.1	0.1	
		500		5:5 *	nr	nr	nr	nr	44.6	nr	50.8	3.9	2.1	1.4	nr	0.9	0.02	
		600		9:1 *	nr	nr	nr	nr	67.2	nr	21.1	3.8	1.9	6.2	nr	2.2	0.2	
		600		7:3 *	nr	nr	nr	nr	59.8	nr	31.7	2.7	1.7	6.0	nr	1.0	0.1	
		600		5:5 *	nr	nr	nr	nr	45.1	nr	42.6	3.0	1.7	0.4	nr	0.8	0.01	
	**MSS + Wood Sawdust (WS)**																	
	MSS	550		5:0 *	31.3	nr	nr	nr	78.4	nr	14.4	0.9	2.0	2.4	2.0	0.7	0.1	[23]
	MSS + WS	550		4:1 *	14.7	nr	nr	nr	69.0	nr	21.3	0.9	1.9	5.1	1.9	0.5	0.2	
	**MSS + Salix (SX)**																	
	MSS	500		100:0 ^nr^	nr	nr	nr	20.4	61.9	17.7	30.1	1.2	3.9	1.9	0.9	0.5	0.1	[25]
	MSS + SX	500		75:25 ^nr^	nr	nr	nr	19.3	54.8	25.5	37.6	1.4	3.7	1.6	0.7	0.4	0.03	
		500		50:50 ^nr^	nr	nr	nr	19.2	41.5	39.3	46.4	1.6	3.2	6.7	0.5	0.4	0.1	
		500		25:75 ^nr^	nr	nr	nr	18.1	30.1	51.8	60.3	2	2.3	5	0.3	0.4	0.1	
	**MSS + Pinewood Sawdust (PS)**																	
	MSS	500		100:0 ***	42.9	nr	nr	19.0	69.3	11.7	18.5	1.0	2.7	7.4	1.2	0.6	0.3	[26]
	MSS + PS	500		50:50 ***	30.7	nr	nr	19.7	50.7	29.6	37.0	1.4	1.5	8.0	0.9	0.5	0.2	
	**MSS + Walnut Shell (WS)**																	
	MSS	600		1:0 ^nr^	31.4	nr	18.1	nr	nr	nr	10.9	1.1	0.7	42.9	1.1	1.2	3.0	[27]
	MSS + WS	600		3:1 ^nr^	47.1	nr	14.2	nr	nr	nr	21.0	1.2	0.9	38.2	0.9	0.7	1.4	
		600		1:1 ^nr^	75.5	nr	7.4	nr	nr	nr	32.9	1.2	1.0	32.1	0.6	0.4	0.7	
		600		1:3 ^nr^	122.9	nr	4.0	nr	nr	nr	47.9	1.4	1.0	26.8	0.4	0.3	0.4	
	**MSS + Cotton Stalk (CS)**																	
	MSS	300		1:0 *	15.6	0.1	16.0	nr	nr	nr	25.2	2.2	3.2	nr	nr	1.0	nr	[28]
		400		1:0 *	16.3	0.1	15.0	nr	nr	nr	24.3	1.7	3	nr	nr	0.8	nr	
		500		1:0 *	9.4	0.1	20.0	nr	nr	nr	20.2	1.1	2.4	nr	nr	0.6	nr	
		600		1:0 *	24.7	0.1	9.8	nr	nr	nr	22.6	0.4	1.3	nr	nr	0.2	nr	
	MSS + CS	300		1:1 *	14.4	0.1	13.8	nr	nr	nr	42.5	3.5	3.6	nr	nr	1.0	nr	
		400		1:1 *	15.8	0.1	12.8	nr	nr	nr	35.2	2.2	3	nr	nr	0.7	nr	
		500		1:1 *	10.8	0.04	14.3	nr	nr	nr	33.6	1.8	2.3	nr	nr	0.7	nr	
		600		1:1 *	22.3	0.1	10.1	nr	nr	nr	32.2	1.7	2.1	nr	nr	0.6	nr	
	**MSS + Rice Husk (RH)**																	
	MSS	550		5:0	31.3	nr	nr	nr	78.4	nr	14.4	0.9	2.0	2.4	2.0	0.7	0.1	[23]
	MSS + RH	550		4:1 *	16.1	nr	nr	nr	70.7	nr	20.3	0.9	2.0	4.2	2.0	0.5	0.2	
	MSS	400		1:0 *	5.5	nr	nr	nr	nr	nr	21.9	1.9	3.1	8.1	0.9	1.0	0.3	[22]
		700		1:0 *	7.1	nr	nr	nr	nr	nr	32.4	0.6	1.7	4.2	1.1	0.2	0.1	
	MSS + RH	400		1:1 *	4.4	nr	nr	nr	nr	nr	30.1	2.0	2.4	14.0	0.5	0.8	0.3	
		700		1:1 *	10.7	nr	nr	nr	nr	nr	26.7	0.7	1.3	9.7	0.6	0.3	0.3	
	**MSS + Rice Straw (RS)**																	
	MSS	800		10:0 ^nr^	15.7	0.1	28.7	7.2	82.3	10.5	6.9	1.2	1.8	7.5	0.4	1.8	0.8	[29]
	MSS + RS	800		7:3 ^nr^	27.7	0.1	13.0	8.1	72.3	19.6	17.8	1.4	1.9	6.4	0.3	0.7	0.3	
		800		5:5 ^nr^	58.5	0.1	5.2	8.8	64.8	26.5	25.6	1.5	1.7	6.3	0.2	0.5	0.2	
		800		3:7 ^nr^	65.3	0.1	4.6	8.4	53.4	38.3	37.2	1.7	1.5	6.1	0.1	0.3	0.1	
	**MSS + Plastics (PP, PVC, PA6)**																	
	MSS	800		10:0 **	nr	nr	nr	nr	nr	nr	23.0	0.5	2.0	2.7	0.1	0.3	0.1	[30]
	MSS + PP	800		8:2 **	nr	nr	nr	nr	nr	nr	23.3	0.5	2.8	0.6	0.3	0.2	0.02	
	MSS + PVC	800		8:2 **	nr	nr	nr	nr	nr	nr	31.4	0.5	2.6	1.7	0.1	0.2	0.04	
	MSS + PA6	800		8:2 **	nr	nr	nr	nr	nr	nr	25.7	0.4	2.7	2.7	0.2	0.2	0.1	
	MSS	550		5:0 *	31.3	nr	nr	nr	78.4	nr	14.4	0.9	2.0	2.4	2.0	0.7	0.1	[23]
	MSS + PVC	550		4:1 *	2.2	nr	nr	nr	70.1	nr	20.0	1.0	1.8	5.8	1.3	0.6	0.2	
	**MSS + Exhausted Tea (ET) or Kitchen Waste (KW)**																	
	MSS	550		5:0 *	31.3	nr	nr	nr	78.4	nr	14.4	0.9	2.0	2.4	2.0	0.7	0.1	[23]
	MSS + ET	550		4:1 *	22.2	nr	nr	nr	68.8	nr	19.7	0.9	2.3	6.3	2.1	0.5	0.2	
	MSS + KW	550		4:1 *	12.1	nr	nr	nr	73.2	nr	19.2	0.9	2.6	2.0	2.1	0.6	0.1	
	**MSS + Digested Manure (DM)**																	
	MSS	525		100:0 ^nr^	nr	nr	nr	20.2	74	5.7	20	0.9	2.5	75.3	1.3	0.6	2.8	[31]
	MSS + DM	525		50:50 ^nr^	nr	nr	nr	19.5	62.2	18.2	30	1.2	2.4	65.5	0.9	0.5	1.6	
Categories of co-substrates:																		
			Forest				Agriculture				Recycling economy				Food residues			Digestate

nr—not recorded, * *w*/*w*, ** dw/dw, *** wt.%.

## Data Availability

Not applicable.
